# Defining the vital condition for organ donation

**DOI:** 10.1186/1747-5341-2-27

**Published:** 2007-11-19

**Authors:** Rinaldo Bellomo, Nereo Zamperetti

**Affiliations:** 1 Department of Intensive Care, Austin & Repatriation Medical Center, Heidelberg, Victoria – Australia; 2 Department of Anesthesia and Intensive Care Medicine, San Bortolo Hospital, Via Rodolfi, 37, 36100 Vicenza – Italy

## Abstract

The issue of organ donation and of how the donor pool can or should be increased is one with significant practical, ethical and logistic implications. Here we comment on an article advocating a paradigm change in the so-called "dead donor rule". Such change would involve the societal and legal abandonment of the above rule and the introduction of mandated choice. In this commentary, we review some of the problems associated with the proposed changes as well as the problems associated with the current model. We emphasize the continuing problems with the definition of death and the physiological process of dying; we discuss the difficulties associated with a dichotomous view of death; we review the difficulties with non-beating heart donation and emphasize the current limitations of society's understanding of these complex issues. We conclude that public education remains the best approach and that such education should not be merely promotion of a particular ideology but honest debate of what is socially and morally acceptable and appropriate given the changes in vital organ support technology and the need to respect patient autonomy.

## Background

In the last 40 years, transplantation has become an effective therapy for end-stage organ failure. The vast majority of viable organs are retrieved from patients who, according to the dead donor rule, are considered to be dead because of irreversible cessation of either cardiac and respiratory function or all brain function [1]. Unfortunately, the definition of the vital status of organ donors has been challenged both for donation after cardiac death (DCD) [2-4] and for brain death (BD) [5-7]

As for DCD, Verheijde and colleagues now propose a "paradigm change to ensure the legitimacy of DCD practice", which should include societal and legal abandonment of the dead donor rule and mandated choice in order to promote individual participation and to specify personal decision about organ donation [8]. This approach requires careful consideration and discussion.

## Discussion

The proposal of abandonment of the dead donor rule, "a switch in the ethics of organ procurement from donor beneficence to autonomy and non maleficence", is not new. As for BD, Truog proposed redefining the condition permitting the retrieval of vital organs in terminal but still living persons: "This alternative [ethical framework] is based not on brain death and the dead-donor rule, but on the ethical principles of non-maleficence (the duty not to harm, or *primum non nocere*) and respect for persons. We propose that individuals who desire to donate their organs and who are either neurologically devastated or imminently dying should be allowed to donate their organs, without first being declared dead. Advantages of this approach are that (unlike the dead-donor rule) it focuses on the most salient ethical issues at stake, and (unlike the concept of brain death) it avoids conceptual confusion and inconsistencies" [7].

We believe that the societal acceptability of such position remains questionable.

The main problem is that this position is still based on the strict dichotomy between life and death, according to which, if a patient is not "surely dead", then she/he must necessarily be still alive. In this case, the process of organ retrieval is per se the cause of death [9]. But will it ever be possible to consider socially acceptable and legally permissible to "kill" someone, even with her/his own permission, to retrieve vital organs? We do not think so.

The quite universal opposition to euthanasia and legally assisted suicide (the developed countries in which they are not punished as crimes can be counted on the fingers of one hand) shows very clearly how much opposition this approach would meet.

An alternative approach is to abandon the above-mentioned dichotomy and recognise the necessity to make prudent decisions in situations where the border between life and death is presently unknown. Modern technology has already intruded into the process of dying and has changed it dramatically. The traditional concepts of life and death are totally inadequate to describe the new situations created by intensive care medicine.

As for DCD, irreversible asystole (defined as sufficiently prolonged absence of cardiac contraction in DNR patients) *appears *to be a point of no return in the process of dying. Yet, as we already wrote [4], the patient's vital condition in such situation is quite out of our traditional concept of life and death. As minutes pass, more and more cells die, but still the "moment of death" remains unknown. Most probably, such a moment is simply impossible to determine as an absolute value, because different organs (and, most likely, different patients) "die" at different times after cardiac arrest. Declaring death at a moment, which is consistent with the retrieval of viable organs is much more a moral than a clinical decision [10].

On the other hand, the equivalence of BD and death rests upon the theory of the "brain as the central integrator of the body". According to this theory, once the brain is dead the organism becomes a rapidly desegregating collection of organs. Such loss of integration shows the biological death of the patient. "A patient on a ventilator with a totally destroyed brain is merely a group of artificially maintained subsystems since the organism as a whole has ceased to function" [11].

Actually, in BD the cortical and brainstem functions upon which the diagnosis of BD is made have never been shown to be recoverable (even after months or years). Yet, some residual intracranial functions can be retained [12-14]. More important, the overall integration of the organism may be not completely over. It can be sufficiently preserved for prolonged, integrated biological maintenance – at least in some cases [5,6,15-17].

What is the value of this residual biological integration? Is it life? Is it a mere biological fact, quite compatible with the concept of death? We do not know. We believe that nobody will ever really know. As things are now, it is not a matter of science. It is a matter of values.

Actually, we do not know what life and death are, in these extreme situations created by hyper-technological medicine. So, we cannot surely identify a border between them. And a definition (even though largely – but not universally – accepted) cannot be a substitute for knowledge [18]. Our opinion is that the wisest thing to do is to suspend our judgement, until we (society as a whole: clinicians, philosophers, bioethicists, lawyers, patients, lay people) are really able to better understand these situations. In other words, we should clearly separate the clinical data (the irreversible asystole and the loss of cortical and brainstem functions) from the social, moral and legal fact (the death of the patient). Then – as decisions have to be made – we should ask ourselves what is the right behaviour in these situations [18] where (again) we are not able to define the patient's vital status with certainty.

## Conclusion

In view of the above consideration, we believe that the best position is the one expressed in 1988 by the Danish Council of Ethics [19] regarding BD. After adequate community involvement, the final decision was that the traditional cardio-respiratory criterion best fits the widely socially accepted concept of human death. Yet, at the same time, the particular significance of brain death ("a condition ... which absolutely excludes the possibility of stopping the death process") was recognised: every support should be forgone, or only maintained to permit organs retrieval: "such an intervention is cause for the conclusion of the process of death, but is not the cause of death". If we change irreversible asystole with brain death, the whole paradigm is complete.

Thus, we can't say when someone is "dead". We could, however, say that irreversible asystole and irreversible apnoeic coma (brain death in current parlance) are clinically and scientifically useful points of no return, which can be used to guide moral and social decisions and legal norms. The condition they identify is neither life not death; it is something in between, a state artificially created by high technology medicine. However, they carry physiological currency, social acceptance and historical weight. The condition they identify can be considered a sufficiently advanced step in the process of dying so that organ retrieval can be allowed in consenting patients

Here we come to second part of the author's proposal, as knowing the patient's position regarding organ donation is an indispensable step in the process. An exhaustive discussion about mandated choice is supplied by the same authors, which discuss the related bioethical issues (mandated choice could be considered as coercive and intrusive on privacy, and it could disallow consideration of the family's views). Above all, they illustrate the practical possible consequence that a majority of people could opt out, if forced to choose. Although the argument that the rules should be changed is legitimate, we do not support it. We think we need to do a lot more education about organ donation, its value to society and its ethical merit before we even think about changing the rules. We think that trying to change the rules may decrease organ donation rates as the public recoils at the renewed gerrymandering of death by doctors for things to fit into their view of what is right.

But what does public education really mean? Full disclosure of all relevant issues at stake and open discussion of all the related implications, or just promotion of donation cards? As the authors admit, "OPOs [organ procurement organisations] today have focused their efforts on convincing members of the public to become organ donors rather than on providing adequate unbiased information and education about organ donation".

We should not confuse genuine education and improving awareness and attention to public health issues with manipulation of people's ideas and opinions. We need to educate people to what is happening now. Anyone who has had to speak (as we regularly do) to families who have just lost a 20 year-old son and then had to get them to grasp the idea of organ donation and the concepts that surround it, knows that the community is a million miles away from the concept of "changing the rules".

## Competing interests

The author(s) declare that they have no competing interests.

## Authors' contributions

Dr. Zamperetti wrote the first draft, Dr. Bellomo revised it thoroughly. The final manuscript was the result of multiple mutually agreed modifications by both authors.
